# Supporting the Development of Grassroots Maternal and Childhood Health Leaders through a Public-Health-Informed Training Program

**DOI:** 10.3390/ijerph21040460

**Published:** 2024-04-09

**Authors:** Kenzie Latham-Mintus, Brittney Ortiz, Ashley Irby, Jack Turman

**Affiliations:** 1 Department of Sociology, Indiana University School of Liberal Arts, IUPUI, 425 University Blvd., Indianapolis, IN 46202, USA; baortiz@iu.edu; 2 Department of Social and Behavioral Sciences, Richard M. Fairbanks School of Public Health, IUPUI, Indianapolis, IN 46202, USA; 3 Department of Pediatrics, Indiana University School of Medicine, IUPUI, Indianapolis, IN 46202, USA; jaturman@iu.edu

**Keywords:** collective efficacy, grassroots leadership, maternal and childhood health, public health training, social capital

## Abstract

The purpose of this research was to assess leadership growth (i.e., changes in personal capacity and social capital) among women living in high-risk infant mortality zip codes who completed a grassroots maternal and childhood health leadership (GMCHL) training program. We used semi-structured qualitative interviews and thematic analysis. Three major themes associated with the training program experience were identified: (1) building personal capacity and becoming community brokers; (2) linking and leveraging through formal organizations; and (3) how individual change becomes community change. Although many of the grassroots leaders were already brokers (i.e., connecting individuals to information/services), they were able to become community brokers by gaining new skills and knowledge about strategies to reduce adverse birth outcomes in their community. In particular, joining and participation in formal organizations aimed at improving community health led to the development of linking or vertical ties (e.g., “people in high places”). The grassroots leaders gained access to people in power, such as policymakers, which enabled leaders to access more resources and opportunities for themselves and their social networks. We outline the building blocks for supporting potential grassroots leaders by enhancing personal capacity and social capital, thus leading to increases in collective efficacy and collective action.

## 1. Introduction

A popular adage among public health researchers is that your zip code is more important than your genetic code for determining your risk of serious health conditions [1]. In the same vein, there is a growing recognition that addressing the social determinants of health, or environmental conditions, is vital for eliminating health disparities [2]. The social determinants of health reflect the social and economic conditions within one’s community that fundamentally shape exposure to health risk factors (e.g., financial instability, community violence, air pollution, etc.) [2]. Like many other health conditions, maternal and childhood health outcomes are intimately linked to the social determinants of health [3,4].

Within the United States, there are stark racial and class disparities in maternal and childhood outcomes. Black mothers are three times more likely to die during childbirth than white mothers [5]. For those over 30 years in age, Black mothers are four to five times more likely to have a pregnancy-related death. Infant mortality rates are more than twice as high for Black mothers [6]. These racial disparities are even worse in Indiana, which consistently ranks near the bottom of all states for maternal and infant health among Black and white women [6]. These differences are not biological, nor explained by individual lifestyles—they are the result of structural racism and the accumulating insults to health that Black Americans face every day [4,7].

In Marion County, Indiana, a small number of zip codes accounts for the majority of infant deaths among Black women [8]. A similar pattern emerges even in rural, mostly white areas, where a handful of zip codes disproportionately contribute to infant deaths in the area [8]. This trend underscores the importance of the social determinants of health in shaping maternal and childhood health outcomes within communities. Although researchers acknowledge the need to address the social determinants of health to see meaningful changes in population health, these efforts are often stymied by the deeply ingrained social inequalities within our society and existing local policies that reaffirm these inequalities [9,10,11].

Because of these challenges, community members living in disinvested neighborhoods are often left to find their own solutions to their community health problems. Capitalizing on the assets within a community, such as the community members themselves, grassroots leadership has the potential to address the social determinants of health [12,13]. Although the contributions of grassroots leaders are often overlooked, the positive impact of grassroots leadership on women’s health movements, and public health broadly, has been well-documented nationally and internationally [12]. One of the main ways grassroots leaders are able to make change is through collective efficacy. According to Bandura [14] p. 75, collective efficacy refers to “people’s shared beliefs in their collective power to produce desired results” and is more than the sum of each group member’s agency. Collective efficacy requires people coming together and collaborating for a shared purpose to make change [14], yet it is not without its challenges. For example, sustaining beliefs and efforts related to collective action may be difficult if the results are slow to manifest or if group members experience opposition [14].

It is clear that grassroots leadership can be highly successful in promoting collective efficacy and improving community health through collective action, but what is less clear is how public health researchers and policymakers can foster and support nascent leadership efforts. The answer may lie with leadership programs aimed at building personal leadership capacity and enhancing social capital. The Grassroots Maternal and Childhood Health Leaders (GMCHL) initiative is such a program, where local, women leaders were identified and participated in a curriculum based on the Social–Ecological Model (SEM) of Health Promotion [15]. The curriculum stressed ecological approaches, including advocating for local policy change, to address inequities related to maternal and childhood health in their communities. The GMCHL program training lasted four months and addressed the following core areas to build the leadership capacity of each woman: leadership development, community health promotion, the causes and consequences of infant mortality, implementing a health equity approach to address disparities, policy advocacy and development, and using storytelling for social systems change (for a detailed description of the curriculum, see Skinner et al. [13]). The formal curriculum was developed using transformative andragogy practices, which recognizes that adults learn differently than children, and was developed with accessibility in mind [13]. The curriculum consisted of four modules (i.e., leadership development and community health promotion, understanding adverse birth outcomes, health equity, and community and policy development), and initial training took place over four months [13]. Skill development was also included in the curriculum, and grassroots leaders participated in workshops focused on storytelling, policy advocacy, and EvaluLead (for more information on EvaluLead, see Groves et al. [16]). After completion of the initial training program, the grassroots leaders continued to be mentored and participated in GMCHL events. 

The most successful grassroots efforts leverage the social capital of community members, develop collective efficacy, and promote collective action [11,17]. This paper focuses on social capital as the catalyst for broader collective efficacy and action. Although multiple definitions of social capital have been developed, we employ Woolcock’s working definition of social capital, which recognizes that “one’s family, friends and associates constitute an important asset, one that can be called upon in a crisis, enjoyed for its own sake and/or leveraged for material gain” [18] p. 67. Indeed, Woolcock [18] emphasizes that a lack of weak ties and vertical linkages (“friends in high places”) excludes individuals from lower socioeconomic statuses and racial/ethnic minorities from employment, educational/training, and housing opportunities. 

A wealth of empirical data has demonstrated that strong social networks and high levels of social capital are health-protective for individuals and communities [19]. Other research underscores that women, particularly women of color, have fewer weak ties and vertical ties, which puts them at a disadvantage for acquiring good-paying jobs and other opportunities for upward mobility [20]. Moreover, the lack of weak ties and vertical linkages among traditionally marginalized communities creates structural holes within social networks, where vital information does not flow [20,21,22,23]. Brokers, however, are individuals who can fill these structural holes by passing information across social networks [21]. 

We draw on Hawkins and Maurer’s [24,25] conceptualization of forms of social capital. Bonding social capital refers to strong ties among actors with similar backgrounds, whereas bridging social capital refers to “relationships amongst people who are dissimilar in a demonstrable fashion, such as age, socio-economic status, race/ethnicity and education” [24] pp. 1779–1780. Bridging social capital connects people across social groups and communities. Finally, linking social capital, also called leveraging social capital, is “the extent to which individuals build relationships with institutions and individuals who have relative power over them (e.g., to provide access to services, jobs or resources)” [24] p. 1780. We are keenly interested in bridging and linking social capital among grassroots leaders. Grassroots leaders often become brokers who allow information and opportunities to flow across diverse social networks [19,20]; however, this requires that grassroots leaders have access to vertical links.

The purpose of this research was to assess changes in personal capacity and social capital in relation to collective efficacy/action among the GMCHL participants. We used semi-structured qualitative interviews and asked the grassroots leaders about changes to their personal capacity and social capital. We paid particularly close attention to the different types of social capital (i.e., bonding, bridging, and linking) that characterized the strength and social distance among social ties [19]. Finally, we explored how personal capacity and social capital were connected to collective efficacy and action. 

## 2. Materials and Methods

### 2.1. Study Setting and Sample

Women were recruited into the GMCHL training initiative from one of the 29 zip codes identified by the state of Indiana as high-risk for infant mortality because of their persistently high infant mortality rates. The GMCHL participants were recommended for the program by a variety of trusted community contacts, including faith-based organizations, social service agencies, affordable housing communities, corrections, and recovery programs. The research team met with contacts within the agencies and discussed the GMCHL training program. The research team explicitly asked the agency contacts for recommendations of women who were not already serving in major leadership roles. Instead, the goal was to connect with women who were passionate about improving birth outcomes in their community and demonstrated compassion, community wisdom, and leadership potential. Recruitment took place from August 2018 to August 2019. All participants were over 18 years of age, lived within a targeted zip code, demonstrated leadership potential according to the source that referred them, and expressed an interest in addressing infant mortality. Most of the women had experienced infant loss directly or indirectly (e.g., sister, aunt, daughter) or had experienced another type of adverse birth outcome. This research was approved by the Indiana University Institutional Review Boards (IRB Protocol # 1708796179). Written consent was obtained from participants. Throughout this manuscript, pseudonyms are used, and identifying information has been removed. 

Each grassroots leader provided 12 h of community leadership per month and received a monthly USD 300 gift card for her efforts. This value was determined by working with women in the community as well as university IRB staff. It was determined that USD 300 gift cards did not interfere with the delivery of social benefits that the women were receiving, and it was a value that the IRB was comfortable with as they believed it did not amount to coercion to participate. Grassroots leaders received a gift card each month that they were active in the program, beginning with enrollment, inclusive of training, and following training if they continued in community leadership work. Grassroots leaders were expected to engage in community leadership work 12 h per month, which could include developing a community program, going to a training session with GMCHL staff, assisting with data analysis or collection, or other activities aimed at improving maternal and childhood health in their community. We adhered to the Kellogg Report on Grassroots Leadership Development [26] and fostered each woman’s personal development while training and mentoring her in community development and advocacy. During November and December 2020, 11 women out of the original 13 who had completed the initial GMCHL training program, led by J.T.J. and A.I., and were still being mentored in their community development work were interviewed. Two grassroots leaders were not interviewed because of scheduling conflicts during the interview period, which were exacerbated by the COVID-19 pandemic. However, both grassroots leaders were still active in the program, and their information was included whenever possible in the analyses. This group of women represents nine of the high-risk zip codes. Table 1 provides an overview of the demographic characteristics associated with the grassroots leaders who were interviewed. 

A semi-structured interview guide (see Appendix A) was developed to capture multiple aspects of personal capacity, social capital, and collective efficacy. Additional information came from the training team and records of community involvement. GMCHL program staff met with the grassroots leaders at least monthly to provide connections to maternal and childhood health (MCH) decision makers (i.e., people and organizations with the power to influence policies that affect maternal and childhood health). Following the four-month initial training program, which consisted of weekly meetings and a public-health-informed curriculum (see Skinner et al. [13] for an overview of the program), the GMCHL program staff held monthly meetings focused on skill building and networking. However, support and flexibility (e.g., adjusting the 12 h service commitment, allowing for breaks from the program, and helping to connect leaders to services) were extended to each grassroots leader to ensure their success in the program. As part of the mentoring and record-keeping process, information about the grassroots leaders’ network connections and work in the community was documented. Grassroots leaders were given the opportunity to review the list of connections and community activities. 

### 2.2. Interview Procedures

Semi-structured interviews were conducted with eleven women using a video conference service (conducted by team member B.O.). The interview guide was informed by previous research into social capital. To illustrate, some questions were adapted from Dudwick and colleagues [27], which provided examples of questions for multiple dimensions of social capital. Three team members (i.e., K.L-M., A.I., and B.O.) developed the interview guide. The interview guide comprised four domains: (1) The Community Context; (2) Access to Groups and Networks Before and After GMCHL; (3) Bonding, Bridging, and Linking Ties; and (4) Community Leadership and Final Thoughts. Interviews were completed using video conferencing software, and audio from the interviews was transcribed. 

### 2.3. Data Analysis

Following Vaismoradi and Turunen’s [28] description of qualitative, descriptive analyses, we employed thematic analysis to analyze the data. Two members of the research team (i.e., K.L-M. and B.O.) coded the interviews using a combination of sensitizing concepts from the literature (e.g., personal capacity, bonding, bridging, linking, brokering, and collective efficacy) and open coding. Examples of subthemes generated from open coding included “the downside of social capital” and “inadequate institutional supports.” Multiple meetings were held to go over coding procedures. After generating our set of codes, we developed broad themes that represented our main findings. To assist with analysis, all transcripts were coded in Word. Codes and corresponding quotes were transferred to an Excel spreadsheet so that team members could easily sift and sort through codes. From the original set of codes, team members (i.e., K.L-M. and B.O.). generated broad themes with a focus on understanding the interconnections among our key concepts. According to Vaismoradi and Turunen [28], a key distinction between thematic and content analysis is interpretation and moving beyond the categorization of qualitative data. We attempted to move beyond categorizing or counting qualitative data by understanding the processes that link personal capacity, social capital, and collective efficacy. 

## 3. Results

### 3.1. Theme 1: Building Personal Capacity and Becoming Community Brokers

Our first major theme was related to personal capacity and becoming community brokers. We purposely emphasize “community” because it was clear that the women who joined the GMCHL program were already serving as brokers to friends and family. This group of women was highly motivated and often sought out opportunities to give back to their community. However, each grassroots leader noted how the GMCHL program had a positive impact on them. The majority of the grassroots leaders highlighted increases in confidence and self-esteem that came from their training. For example, Nancy highlights how she has become more comfortable communicating with strangers.

*…I’m able to communicate when I have a new connection even if it’s an acquaintance, before, two years ago when I wasn’t a grassroots leader yet, I had a very hard time to communicate with anyone. So a gas station trip was a lot for me. So now I’m completely opened up to things.—Nancy*

Ivy expressed a similar sentiment about being able to be more comfortable with communication and being outgoing, despite being shy.

*I think I’m more outgoing than I was. I am still kind of shy on talking to people. But I’m more outgoing and speaking up.—Ivy*

Jordon connected her leadership training to her work experiences and noted that she felt more confident.

*…I feel like I’m more confident and in my work and my interactions with other people, I think that it’s made me more confident in my relationships. And I feel like I’m a much better communicator now because of grassroots. And I feel like my relationships are stronger because of my grassroots experience for sure.—Jordon*

Others noted tangible skills and knowledge that they had honed during their time as a grassroots leader, such a public speaking, storytelling, conducting research, and safe sleep practices. To illustrate, Brenda highlighted how the training provided the grassroots leaders with concrete skills.

*Well, in the program, there are several things that—requirements there has to be done. And they take you through training, get training on how to speak publicly, how to do research, how to research different things and compile a list of things to do and, and get it done.—Brenda*

Additionally, Lisa articulated how empowering the program had been for her and how she planned to share these skills and knowledge with her children.

*So being a grassroots maternal and child health leader has—the biggest thing I can say is empowered me and given me the skills and the confidence to speak to people, it’s given me the motivation—when they share those data and statistics about how moms and babies died and now that I have an understanding of social determinants of health and I have an understanding of systematic oppression, historic trauma, and the things that plagued my neighborhood and plague my community and plague my family…I will always be an advocate and I’m trying to pass it onto my children as well, you know, it has definitely made me commit to being part of the change that I want to see and definitely helped made me see that I have the power to do so. [emphasis added]—Lisa*

This quote from Lisa also emphasizes how the Social–Ecological Model (SEM) of Health Promotion curriculum helped grassroots leaders make the connections between systems of oppression and the public policies that have shaped their communities. 

Because the grassroots leaders had first-hand experiences with infant mortality, many described the training as a way to translate their loss into a sense of purpose. Brenda explained how the loss of her grandson motivated her participation in the GMCHL program, and how she developed a passion for safe sleep.

*My passion is with safe sleep with babies. A lot of people are not aware of different things—the do’s and don’ts to prevent infant mortality. That, I, even as when I had my kids years ago, things that I did, but I was just blessed and it didn’t happen to me, but it happened to my daughter with my grandson. And now, I’ve been taken a class on different things, the do’s and don’ts that you wouldn’t even think of. You think you’re doing it out of love. But it’s really harmful to the baby and can cause death.—Brenda*

Kayla described how the GMCHL program helped her heal and allowed her to share her life story.

*It afforded me to get some healing from the shame and the guilt. Not only every time I shared my story. I shared it like twice in the grassroots like going to different places sharing it with people I get a little healed by that. A little bit of the shame a little bit of the guilt continues to go away and I give some hope to them.—Kayla*

Other leaders described how the program was a way to give back to the community and improve outcomes for current teens and young adults in their community. Donna described her desire to help the “future me’s:”

*And what I’m doing. As far as, what I’ve done, I’ve spoken up for people like me or the future “me’s” out there like the kids who will later be young, pregnant and not know what to do.—Donna*

Through training, the grassroots leaders acquired important skills, which gave them increased confidence to share their stories and provide information and emotional support. Moreover, for some women, becoming a grassroots leader led to a greater sense of purpose, healing, and the chance to give back to the community and help future generations. 

The process of building personal capacity allowed the grassroots leaders to become community brokers within their communities. For example, Brenda describes how she now shares information with her neighbors.

*[I’m] able to share some information with the neighbors there that say the young girls in an area that might be pregnant, I’m able to reach out to them and share different information…Some, just share with them on safe sleep practices…I’m able to tap into the neighborhood. My neighbors, friends, and family, really, share with them the things that I do and different resources that they’ll be able to share with them. And that’s something I was never, I wasn’t able to do that before.—Brenda*

Nancy describes not only trying to provide information to community members, but also providing instrumental support by connecting new mothers to people whose babies or children had outgrown items.

*As far as my immediate community. I try to look out for the mothers that have children that I know I can give something or get something or you know, like from one person to another, that’s out grown these shoes or this stroller. I think I’ve tried to connect things that other people don’t need to people that do and it’s kinda comes, it’s making a full circle.—Nancy*

By raising their own personal capacity, the grassroots leaders became community brokers. Many of the women who decided to participate in the program were already serving as brokers to their friends and family or were considered the “go-to” person for information or assistance, yet they still perceived the GMCHL program as enhancing their knowledge and skills. This was particularly true in relation to safe sleep practices. For example, Kyra remarked that people already sought her out for help, but she had become more of a resource since joining the GMCHL program.

*Um, I don’t think they’ve changed much. In terms of like, people seek me out for resources and advice. Like when it comes to infants and babies. Like I just had a friend who had her first child, so she asked me for information like, you know like, what should she should be looking for. I’ve become more of a resource, I would say.—Kyra*

Andrea highlighted how she has become a critical source of information about safe sleep practices in her community.

*It’s actually been amazing. I spread awareness to everybody and everybody who have question about co-sleeping. They will come and talk to me about the proper way to co-sleep and the danger of co-sleeping.—Andrea*

With their training, the grassroots leaders felt more knowledgeable and were eager to take that knowledge back to their social networks and communities. 

Although every grassroots leader described how the GMCHL program positively affected them, it is worth noting that two grassroots leaders hinted at the potential dark side of being a community broker. Lisa and Donna both discussed being overwhelmed with their commitments to formal organizations. Both grassroots leaders were highly active in organizations and expressed that having multiple obligations was difficult to manage. In the case of Lisa, she recognized that she was overcommitted, and she was in the process of scaling back her commitments: “I have so many things on my plate. I’ve been trying to take things off instead add things on.” When asked about how their immediate social networks had changed, a few women also mentioned downplaying or keeping their activities and work with the GMCHL program from their families for fear of putting distance between them. These issues underscored that increasing social capital may accompany unintended, potentially negative, consequences. 

### 3.2. Theme 2: Linking and Leveraging through Formal Organizations 

Our second theme was related to linking and leveraging through participation in formal organizations. Nearly all of the grassroots leaders discussed positive changes to their social network, which included elements of bonding, bridging, and linking. As to be expected, bonding was most commonly described, where grassroots leaders focused their energies on their stronger ties, including family, friends, and neighbors. However, six grassroots leaders explicitly stated that their social networks had expanded beyond close friends, families, and neighbors. In these cases, the grassroots leaders began to tap into weaker ties. Moreover, some women discussed having connections to people they normally would not have known (e.g., social workers and nurses). 

However, the major impetus for vertical linkages came through formal organizations. Out of the eleven leaders interviewed, ten had started volunteering and/or working (e.g., full-time, part-time, or consultant work) at new organizations aimed at improving the health and well-being of the community. More specifically, eight grassroots leaders had received paid positions, which allowed them to sustain their leadership work by being embedded in agencies, non-profits, or industry work that advance maternal and childhood health. Prior to joining the program, only two grassroots leaders were part of formal organizations that were focused on community issues. Therefore, we can see a large increase in the breadth and depth of community involvement from the grassroots leaders. Multiple grassroots leaders began working with state- and county-level health departments and programs, while others became affiliated with non-profits focused on a range of issues, such as community health, housing, and employment.

Much of the facilitation between the grassroots leaders and formal organizations came through the research team. As a strategic component of the GMCHL program, its existence was marketed to state and county agencies and non-profits. Using their own social capital as a researcher and faculty member, the research lead made connections with other MCH decision makers. The research lead served as a bridge between the grassroots leaders and the initial organizations and advocated for the grassroots leaders. It was stressed to MCH decision makers located in state or county government agencies and non-profit organizations that they needed grassroots leaders on their teams to fully understand community needs and solutions. Following these conversations, the MCH decision makers wanted to meet the women and hear their stories and opinions. After participating in a few key events and sharing their stories and insights, additional community organizations invited the grassroots leaders to participate. Success in one program or organization led to more opportunities and interest from MCH decision makers. 

After joining these formal organizations, the grassroots leaders’ social networks expanded rapidly, including increases in linking ties. Kyra summarized her experiences with a growing and diversifying social network.

*My network is definitely a lot bigger. And, it is a mix of people—who, like, the social stratification divide—it’s like a wide range of people. Like people who have may not have any income and are completely disenfranchised to people who are CEOs at the company and stuff like that. It’s definitely expanded my social circle.—Kyra*

Donna, who was already involved with other organizations prior to the GMCHL program, described the changes to her social network, including knowing politicians.

*Joining the program has been very—like my social network is, it’s pretty broad. Now. I can actually say that I know people in politics. I can talk to people who deal with politics about, you know, the things within my community that I know them directly in that I know how to get in contact with them more readily than I would have.—Donna*

Through these affiliations, the grassroots leaders made new connections, often reflecting vertical linkages. Not only did their social networks expand, but we were able to document through their experiences evidence of enhanced bridging and linking social capital. 

### 3.3. Theme 3: How Individual Change Becomes Community Change 

Our third and final theme described the connections among individual change, collective efficacy, and collective action. After having the grassroots leaders detail changes to their social networks, we asked them to discuss what actions they had taken since joining the GMCHL program to make changes to their communities. As part of their training, grassroots leaders were expected to engage in work that raises the community consciousness regarding infant mortality prevention and help work to bring about systemic change in their community, a local organization, and/or a government agency. When asked about their community involvement, most grassroots leaders mentioned the activities that they had completed with other grassroots leaders, such as hosting community baby showers. For example, Jordan described her involvement with the community baby showers and other GMCHL activities.

*We’ve participated in, like, community baby showers. We held—we did a whole safe sleep meeting. I get they got the—what we call it, like a focus group. I have spoke different places about well, I’ve spoke to people about grassroots, but I’ve also spoke to people about safe sleep. And let’s see we’re working on making YouTube videos for focused on teens and…the YouTube videos are going to focus on basic child care, like how to change a diaper, how to prepare a bed, different things like that, but also self-care and how moms need to take care of themselves so that they can take care of their babies.—Jordan*

Others discussed activities that they had participated in through their organizational affiliations. Kyra described some of the activities she had participated in as a member of a local chapter of a national community advocacy group.

*We provide community resources, to help those who are disenfranchised in the community. We advocate for education and provide information on dealing with courts and addiction, and housing and education. And making sure [school children] have bookbags and supplies things like that.—Kyra*

Not only did the grassroots leaders come together for a shared purpose (collective efficacy) among themselves, but they also participated in larger collective action efforts in the community. Furthermore, it was clear that many of the leaders also understood the need to come together to make change and the potential of grassroots movements. Lisa nicely summarized this idea.

*You know, I didn’t like what was going on in my community, but I didn’t know how to change it. So I was just in the mode of living, surviving—trying to pay my bills, raise my children, focus on my life. So now that I am in advocacy, the Grassroots and Maternal and Child Health Leadership, it has empowered me to show my stories have power that can affect change at the political level. Stories can affect change in my community where I developed relationships that help people think about their life and possibly want to change them in the right direction.—Lisa*

Lisa’s quote also underscores one of the most critical points about grassroots leadership—its sustainability. Grassroots leaders beget other grassroots leaders. The influence is far-reaching, possibly spanning over generations, as expressed by multiple leaders who wanted to share their knowledge and passion with their own children and grandchildren as well as the teens and young adults in their communities. It is the type of long-term advocacy and planning that is needed to address the most pressing health concerns within any community.

Among the original 13 grassroots leaders, every leader participated in some level of community education, advocacy, or development work. Although some leaders were more active than others, each grassroots leader participated in collective action efforts that reached beyond their immediate network. As a group, the grassroots leaders have given multiple presentations across the community. They have shared their stories and knowledge with government officials, public health advocates, and scholars. They have conducted focus groups and collected other data to inform interventions. They have written op-eds and created workshops to disseminate their knowledge about their communities’ risk of infant mortality. They have spoken with local, state, and national government officials and provided feedback on specific bills and initiatives aimed at improving maternal and childhood health. Furthermore, the grassroots leaders have participated in the development of new programs, such as a community transition program for mothers in prison and introducing a maternal mortality review committee process to collect family narratives. The collective action taken by the grassroots leaders has led to real changes in policies around the community.

**Figure 1:**
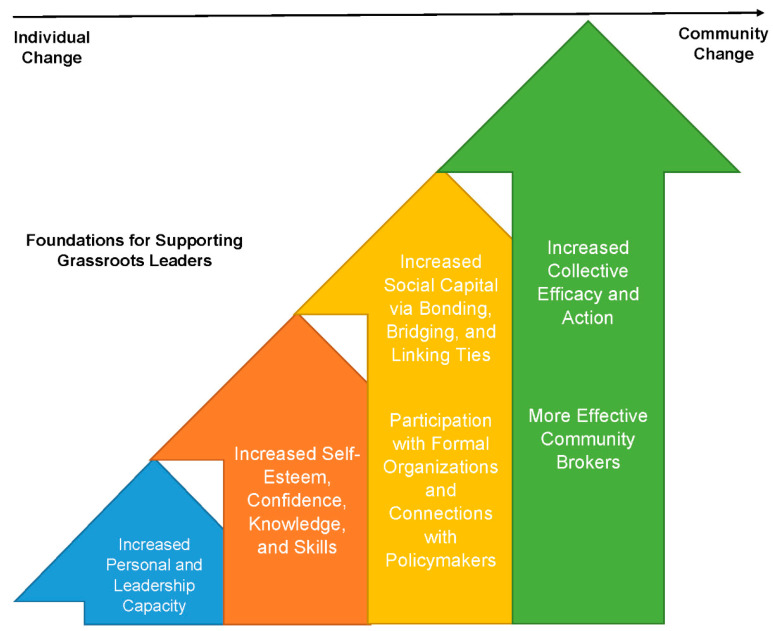
Foundations for supporting grassroots leaders.

Based on the above themes, we created a conceptual framework that links individual change to community change. In Figure 1, we outline the building blocks for supporting the potential of grassroots leaders. Through formal public health training, the grassroots leaders grew their personal and leadership capacity, which led to an increase in self-esteem, confidence, knowledge, and skills (e.g., public speaking). With their newly acquired skills, the grassroots leaders increased their social capital. In particular, with facilitation from the research team, the grassroots leaders were able to develop linking ties by participating in formal organizations and making connections with policymakers. The outcome of increased social capital was increased collective efficacy and collective action.

## 4. Discussion

The purpose of this research was to assess whether participation in a grassroots leadership program (i.e., GMCHL) increased personal leadership capacity, enhanced social networks, particularly linking bonds, and led to collective efficacy and action among participants. Based on semi-structured interviews with the grassroots leaders, it is evident that the leaders felt that their personal capacity had grown. However, many of the grassroots leaders were already active within their social networks. This echoes previous research that suggests that grassroots leaders report more self-efficacy and optimism relative to other community members [29]. The GMCHL program was able to nurture the budding leadership qualities that existed within each participant. Community development and resident mobilization are often difficult and time-consuming, with the lack of skilled leaders being an important barrier to sustained efforts [30]. The women who participated in the GMCHL were already leaders within their own social networks; however, the program was able to provide opportunities to develop the skills and knowledge needed to grow their broader community and systems’ leadership potential.

Additionally, the grassroots leaders reported increased social capital, including changes to their bonding, bridging, and linking ties. Through thematic analysis, we observed that much of the linking social capital occurred through participation in organizations aimed at improving community health. The majority of the grassroots leaders gained new affiliations with public health programs, community advocacy groups, and other local organizations. These experiences allowed the grassroots leaders to connect with MCH decision makers (e.g., policymakers, CEOs, and professionals working in the area of maternal and childhood health). Not only did these affiliations create linking ties, but the organizations also benefitted from having local grassroots leaders who are intimately aware of the problems within their own communities on their boards and advisory councils. We believe this finding reiterates the role of public health interventions aimed at increasing social capital. Connecting highly motivated grassroots leaders with a curriculum that enhances personal capacity is only the first step. To have far-reaching, meaningful changes in access to resources, such as information and opportunities among community members, grassroots leaders need to become effective brokers who are able to fill structural holes and pass information across social networks [20,21].

The primary mechanism for these changes in social capital came through opportunities to join formal organizations. The research team leaders used their connections as public health scholars and community advocates to facilitate many of these opportunities. In designing this program to help bring about sustainable changes in social, economic, and political systems that underlie inequities in birth outcomes, the researchers realized that it was important to establish efforts, like the GMCHL initiative, to bring together the social networks of the leaders with those of local and state MCH decision makers.

Although team leadership facilitated network opportunities among the grassroots leaders to share the realities of adverse birth outcomes in their own lives or in their communities, connections among organizations and MCH decision makers were prioritized based on the perspectives of the grassroots leaders regarding the inequitable systems (either government or organizational) and what needed to be addressed to improve birth outcomes. There was a consensus that the state health department was a priority, and likewise the leadership at the state health department was interested in growing relationships with the grassroots leaders. Other examples of priorities generated by the grassroots leaders included connecting with organizations that focused on housing [31], the justice system [32], and faith-based organizations [33] to reach women in the community who were at risk of adverse birth outcomes. When MCH decision makers heard the grassroots leaders’ stories and realized the powerful connections that the grassroots leaders have with their communities [34], the MCH decision makers began to invite, and continue to invite, grassroots leaders to participate in decision-making committees or panels. Although network connections and opportunities were facilitated by the GMCHL research team and program staff, the grassroots leaders had a large role in driving the direction of the initiative. The leaders identified the areas of need in their community and worked to develop solutions. Furthermore, each grassroots leader was able to choose their leadership focus, and they were able to use their newly gained skills and social capital to drive policy changes at the local and state level. This is an important change to MCH decision making in our state. These initial outcomes occurred because connections were made among academics and community members to grow skilled grassroots leaders.

Although we were able to document changes in personal capacity and social networks among the grassroots leaders and specific examples of collective action, it is difficult to capture the long-term impact of this program. The ultimate goal of the GMCHL program is to improve infant and maternal health outcomes in high-risk communities by addressing the social determinants of health through grassroots leadership. This is a lofty goal that will take time and investment from multiple stakeholders. Yet, there was *clear* evidence of collective efficacy and collective action already taking place. Some of these efforts were directly tied to the GMCHL program, whereby grassroots leaders fulfill expected responsibilities to create public health interventions in their communities. However, other grassroots leaders began to participate in other advocacy efforts through their participation in local organizations, suggesting that community leadership efforts transcend the program. This research speaks to the potential of grassroots leadership to promote community- and systems-level change. Nonetheless, we acknowledge that the sustainability of such efforts requires long-term commitment from grassroots leaders and research team members. Thus, it is hard to predict the impact of this program multiple years into the future; however, by increasing personal and leadership capacity, making in-roads with local MCH decision makers, and demonstrating the success of collective efficacy and action in communities disproportionately impacted by infant mortality, we hope that this program becomes self-perpetuating.

## 5. Conclusions

Based on the perceptions of the grassroots leaders, the GMCHL program has been successful in its goal of raising the personal leadership capacity of grassroots leaders living in high-infant-mortality communities. Long-term success in addressing racial and social class inequities in maternal and childhood health outcomes will take sustained grassroots efforts. The findings from this research underscore the role of public health interventions in supporting the development of grassroots leaders. The grassroots leaders were able to become community brokers by gaining new knowledge about safe sleep practices, infant mortality rates in their community, formal public health practice, and taking a social ecological approach to reduce adverse outcomes in their community. They also gained access to people in power, such as policymakers, which enabled leaders to access more resources and opportunities for their social networks.

## Figures and Tables

**Table 1:** Interviewee characteristics.

Characteristics		*N (%)*
Age Range	25–39	4 (36%)
	40–54	3 (27%)
	55–70	3 (27%)
Annual Income	USD 0–10,000	5 (45%)
	USD 10,001–25,000	2 (18%)
	USD 25,001–50,000	2 (18%)
	USD 50,001–75,000	2 (18%)
Education Level	Graduated high school/GED	3 (27%)
	Attended some college, no graduation	1 (9%)
	Obtained technical certification	1 (9%)
	Associates Degree	2 (18%)
	Bachelor’s Degree	2 (18%)
	Master’s Degree	1 (9%)
Employment Status	Unemployed	2 (18%)
	Self-employed	1 (9%)
	Part-time	4 (36%)
	Full-time	3 (27%)
	Retired	1 (9%)
Relationship Status	Single	3 (27%)
	Domestic partner	3 (27%)
	Married	3 (27%)
	Divorced/Separated	2 (18%)
Number of Children	1–3	9 (82%)
	4 or more	2 (18%)
Race	White	3 (27%)
	Black	8 (73%)
Rural vs. Urban	Rural	2 (18%)
	Urban	9 (82%)

## Data Availability

Data are unavailable for public use.

## Appendix A

*Domain #1: Community Context*

- What are the most pressing needs within your community?
- What changes or improvements would you most like to see in your community?
- What are the resources (e.g., programs, services, transportation, people, places, technology, etc.) available within your community? Does everyone have access to, or can get these resources? If not, who does or does not have access?
- Has the Grassroots Maternal and Childhood Health Leadership program helped you to identify these needs and resources?
- If yes, can you give a specific example?

*Domain #2: Access to Groups and Networks before and after* *GMCHL*

- Think about your own social network and relationships prior to participating in the Grassroots Maternal and Childhood Health Leadership program.
- Did you belong to any groups such as church, boards, community groups, organizations, or associations before becoming a grassroots leader? If so, please describe the goals the group or organization?
- Since joining the Grassroots Maternal and Childhood Health Leadership program, do you belong to any *new* groups, organizations, or associations? If so, please describe the what the group or organization does?
- Since joining the Grassroots Maternal and Childhood Health Leadership program, how have your personal relationships changed (i.e., family and friends)?
- How has that changed your life?
- Since joining the Grassroots Maternal and Childhood Health Leadership program, how has your community relationships changed (i.e., people in your community, neighbors, co-workers, or acquaintances)?
- How has that changed your life?

*Domain #3: Bonding, Bridging and Linking*

- Next, I am going to ask you about different situations where someone in your community needs help.
- A neighbor or friend is struggling to pay a major bill or expense. Who would you contact (or tell them to contact) to try to help your neighbor get the money they needed?
- Why this person/contact? How do you know this person/contact?
- A neighbor or friend is facing eviction after not being able to pay their rent. Who would you contact (or tell them to contact) to try to help your neighbor avoid eviction?
- Why this person/contact? How do you know this person/contact?
- A neighbor or friend does not have enough to eat. Who would you contact (or tell them to contact) to help them get access to food.
- Why this person/contact? How do you know this person/contact?
- A neighbor or friend is currently pregnant and is having trouble getting access to medical care. They currently do not have health insurance and are worried about the cost of care and transportation. Who would you contact (or tell them to contact) to help them get access to medical care?
- Why this person/contact? How do you know this person/contact?
- A neighbor or friend has been unemployed for an extended period. They have been applying to many jobs without success. Who would you contact (or tell them to contact) to try to help find employment or training opportunities for your neighbor?
- Why this person/contact? How do you know this person/contact?
- A teen is thinking about going to college but is overwhelmed by the application process. Who would you contact (or tell them to contact) to help them with their applications?
- Why this person/contact? How do you know this person/contact?
- A neighbor or friend wants to get involved and work toward reducing crime in the community. Who would you contact (or tell them to contact) to help them get more involved.
- Why this person/contact? How do you know this person/contact?
- A neighbor or friend wants to get registered to vote in the community. Who would you contact (or tell them to contact) to help them get more involved.
- Why this person/contact? How do you know this person/contact?
- A neighbor or friend is thinking about running for a local election, but wants to speak with someone who has gone through the process first. Who would you contact (or tell them to contact) to learn more about running for an elected position.
- Why this person/contact? How do you know this person/contact?
- Looking at all the resources or contact you mentioned how did you come to find them?

*Domain #4: Community Leadership and Final Thoughts*

- Has the Grassroots Maternal and Childhood Health Leadership program changed how you think about leadership and community advocacy? If so, please explain.
- Describe what you have done to help with the needs of your community? How has your social network (groups, organizations, people you know) including other Grassroots Leaders helped you to advocate for your community?
- Do you have any other thoughts or experiences that you would like to share regarding the Grassroots Maternal and Childhood Health Leadership program and its effects on your social networks and ability to make change?
- Some questions were adapted from Dudwick, N., Kuehnast, K., Jones, V. N., and Woolcock, M. (2006). Analyzing social capital in context. A guide to using qualitative methods and data, 1–46.
